# Supplementary material for: Comparative assessment of single-stage and two-stage anaerobic digestion for biogas production from high moisture municipal solid waste
Source: PeerJ. 2020 Aug 19;8:e9693. doi: 10.7717/peerj.9693 (PMC7443091; doi:10.7717/peerj.9693)
Supplement: Supplemental Information 3 [file peerj-08-9693-s003.docx]

| Composition of municipal solid waste | Percentage by weight |
| --- | --- |
| Fruits, Vegetables | 37.32 |
| Paper | 11.52 |
| Plastic | 25.18 |
| Clothing | 9.49 |
| Wood | 4.8 |
| Rubber | 0.79 |
| Glass | 7.63 |
| Metal | 0.73 |
| Stone, Bone Tile | 1.14 |
| Foam | 1.4 |
| Total percentage | 100 |
